# Quantifying 6D tumor motion and calculating PTV margins during liver stereotactic radiotherapy with fiducial tracking

**DOI:** 10.3389/fonc.2022.1021119

**Published:** 2022-11-16

**Authors:** Xingru Sun, Zhitao Dai, Meiling Xu, Xueling Guo, Huanfan Su, Yang Li

**Affiliations:** ^1^ National Cancer Center/National Clinical Research Center for Cancer/Cancer Hospital and Shenzhen Hospital, Chinese Academy of Medical Sciences and Peking Union Medical College, Shenzhen, China; ^2^ Department of Radiation Oncology, The First Affiliated Hospital of Navy Medicial University, Shanghai, China; ^3^ Department of Medical Imaging, Jiangxi Medical College, Shangrao, China

**Keywords:** liver SBRT, CyberKnife, fiducial tracking, tumor motion, PTV margin

## Abstract

**Objective:**

Our study aims to estimate intra-fraction six-dimensional (6D) tumor motion with rotational correction and the related correlations between motions of different degrees of freedom (DoF), as well as quantify sufficient anisotropic clinical target volume (CTV) to planning target volume (PTV) margins during stereotactic body radiotherapy (SBRT) of liver cancer with fiducial tracking technique.

**Methods:**

A cohort of 12 patients who were implanted with 3 or 4 golden markers were included in this study, and 495 orthogonal kilovoltage (kV) pairs of images acquired during the first fraction were used to extract the spacial position of each golden marker. Translational and rotational motions of tumor were calculated based on the marker coordinates by using an iterative closest point (ICP) algorithm. Moreover, the Pearson product-moment correlation coefficients (*r*) were applied to quantify the correlations between motions with different degrees of freedom (DoFs). The population mean displacement ( $\bar{M_{P}}$ ), systematic error (Σ) and random error (*σ*) were obtained to calculate PTV margins based on published recipes.

**Results:**

The mean translational variability of tumors were 0.56, 1.24 and 3.38 mm in the left-right (LR, *X*), anterior-posterior (AP, *Y*), and superior-inferior (SI, *Z*) directions, respectively. The average rotational angles *θ*_*X*_ , *θ*_*Y*_ and *θ*_*Z*_ around the three coordinate axes were 0.88, 1.24 and 1.12, respectively. (|*r*|>0.4) was obtainted between *Y* -*Z* , *Y* - *θ*_*Z*_ , *Z* -*θ*_*Z*_ and *θ*_*X*_ - *θ*_*Y*_ . The PTV margins calculated based on 13 published recipes in X, Y, and Z directions were 1.08, 2.26 and 5.42 mm, and the 95% confidence interval (CI) of them were (0.88,1.28), (1.99,2.53) and (4.78,6.05), respectively.

**Conclusions:**

The maximum translational motion was in SI direction, and the largest correlation coefficient of *Y*-*Z* was obtained. We recommend margins of 2, 3 and 7 mm in LR, AP and SI directions, respectively.

## 1 Introduction

Hepatocellular carcinoma (HCC) is the seventh most common cancer and the third most common cause of cancer-related deaths (1). According to clinical guidelines, there are several treatment options to choose from these including surgical resection, percutaneous and transarterial interventions, liver transplantation, chemotherapy, immunotherapy and radiotherapy. Technological advancements in treatment planning and delivery have made it practical to provide radical doses to the tumor volume while selectively preserving the surrounding normal tissue. In particular, the advent of stereotactic body radiotherapy (SBRT) techniques has enabled a steeper dose fall-off gradient to enhance tumor control probability (TCP) while suppressing the normal tissue complication probabilities (NTCP) (2–6). SBRT, especially with the clinical feasibility of CyberKnife (CK) system has been demonstrated to be a safe and effectiveective noninvasive treatment for HCC in several studies (7, 8).

In implementation of SBRT, amplitude changes of respiratory motion are more important because of higher fractional dose and steeper dose fall-off. Liver movement due to breathing is one of the largest sources of internal organ movement. Target movement up to a few centimeters was obtained for liver, which indicates that liver motion is one of the largest sources of internal organ movement, second only to respiratory movement (9–13). Motion of tumor and adjacent organs during treatment may lead to an insufficient dose of tumors and/or over-irradiation of normal tissues. To ensure target coverage, additional margins are extensively used to mitigate the adverse effects of intra- and inter-fractional organ motions (14, 15), which means that more normal tissues will be included in the irradiation fields. Fortunately, Accurate image guidance makes allows one to define patient-specific CTV-PTV margins possible (16), which may lead to reduction of the normal tissue toxicities (17–19).

Many studies have tried to quantify the margin between the CTV and PTV by using different imaging modalities, including MV portal (20, 21), CBCT (21, 22), 4D-CBCT (9, 23), kV fluoroscopy (13, 24–28), electromagnetic trackers in Calypso (29, 30), orthogonal pair X-ray images for CK (12, 31–35), orthogonal X-raysin ExacTrac (25), ultrasound (36, 37), optical surface imaging (38), MR (39), and multimodal imaging (11). Among these techniques, kV imaging demonstrated either sufficient intra-fraction motion monitoring in liver SBRT (40), or accurate treatment delivery (41). In particular, real-time tumor tracking using an internal fiducial marker integrated into CyberKnife ^®^ (CK) system has been demonstrated high accuracy (42–45). By using different tracking techniques, the CK system can monitor translational and/or rotational target motion with 6 degrees of freedom (DoF) by registering simultaneous orthogonal pair X-ray images to the digitally reconstructed radiographs (DRR) generated from planning CT. For liver SBRT, the fiducial tracking technique is usually applied, and the tumor position variability could be constructed based on the 3D fiducial positions. Different methods such as least squares fitting (12) and iterative closest point (ICP) (33, 34) methods were used to calculate tumor motions.

The planning target volume (PTV) that takes into account the effects of all possible geometric uncertainties is used to ensure the clinical target volume (CTV) is fully covered by the prescribed dose. Lots of different methods can specify the margins required for those uncertainties, among which the extents of inter- and intra-fractional variation are significant factors when evaluating individual and population-based margins calculations. Most the margin recipes were expressed in terms population systematic error (Σ ) and random error (*σ* ) (10, 14, 46–52). Especially, the dose penumbra was also included in the margin formulas (43, 49, 53).

The purpose of this study was threefold (1): to quantify the 6D position variability for liver SBRT with fiducial tracking technique (2), to analyze the correlations between different DoF, and (3) to estimate PTV margins based on published recipes in order to supply PTV margins for potential recommendations to be selected in the clinic.

## 2 Material and methods

### 2.1 Patient selection and data acquisition

Intrafraction kV images have been assessed for 12 patients (58 ± 12 years old, 6 males/6 females, typically 3 to 6 fractions) undergone liver SBRT with CyberKnife robotic radiosurgery system (Accuray, Inc., Sunnyvale, CA, USA) between 2015 and 2018. Before the treatment, three (8 patients) to four (4 patients) fiducials (golden seeds, ~0.7-1.2 mm diameter by ~3.0-6.0 mm length) are implanted inside or adjacent to the tumor in accordance with clinical requirements and fiducial tracking rules. Fiducial placement for soft tissues was expressed as follows:

- Minimum 18 mm spacing between fiducials
- >15^∘^ angle between fiducials
- Ensure all fiducials can be seen in >45^∘^ oblique views with no overlap
- The distance between the geometric centroid of the fiducial set and the geometric centroid of the target must be 50 mm or less.

**Figure 1:**
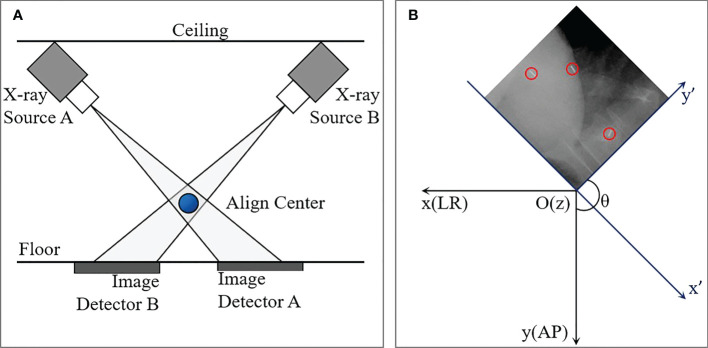
Diagram of the CyberKnife imaging system **(A)** and schematic of coordinate transformation between image coordinate system (*x* ^"^,*y* ^"^) and patient coordinate system (*x*,*y*). The blue ball in **(A)** is the align center. The blue and black axes in **(B)** represent image and patient coordinate systems, respectively. The red circles indicated fiducials detected on kV image.

Before the planning CT scan was executed, those implantation regulations were completed about one week in order to offer an adequate time interval for fiducial stabilization. CK Synchrony fiducial tracking method was used during the whole treatment without respiration restrained. As was shown in **Figure 1A**, the Two orthogonal X-ray sources equipped on the ceiling and two amorphous silicon panel detectors equipped on the floor are the main components of CK image guidance system. 495 pairs of orthogonal KV images of all patients were acquired during the first fraction. All data of patients were collected under the condition with all patients consent and Research Ethics Board (REB) approved local clinical trial.

### 2.2 Fiducial segmentation and 6D tumor position variability construction

For each image, the 1024×1024 voxel matrix was converted to a binary image containing the numbers 0.0 (Black) and 1.0 (white) by setting a gray threshold. The position of a fiducial marker was defined as the mass center of the ‘white bar’, and accordingly the two-dimensional (2D) coordinates on each pair of the orthogonal images were obtained. An example of a kV image with three fiducial markers was displayed in **Figure 1B**, and the red circles indicated fiducials detected on kV image. And then a rotational matrix is used to transform all the positions from the image coordinate system (*x* ^"^,*y* ^"^,*z* ^"^) into the patient coordinate system (*x*,*y*,*z*), where +*X* is in the inferior direction, +*Y* is in the left direction, and +*Z* is in the anterior direction assuming that the patient is lying supine on the treatment couch.

The target shifts in left-right (LR), anterior-posterior (AP), and superior-inferior (SI) directions were calculated separately as the difference between the target position at the beginning of the treatment and the target position at any given point during treatment. The iterative closest point (ICP) algorithm is designed to calculate the translational and rotational movement of the tumors. The aim of the ICP algorithm is to find a rigid rotation matrix *R*_0_ and translation vector *T*_0_ that minimizes the mean square sum of the Euclidean distances between the target point set ***Y*** transformed by (*R*_0_, *T*_0_) and its closest point set ***Z*** in ***X***. A detailed description of the ICP algorithm can refer to the **Supplementary Material**. The translational and rotational movement of the tumor were recorded as (*X*, *Y*, *Z*) and (*θ*_*X*_,*θ*_*Y*_,*θ*_*Z*_) , respectively. Translational motion range in 3D space (*R*) can be calculated from the measurements in X, Y and Z directions, by the square root of the summation of squares, namely $R=\sqrt{X^{2}+Y^{2}+Z^{2}}$ .

### 2.3 Analyzing of tumor motions and population errors

For the *i* th patient, the mean values (*M*_*Pi*_) and standard deviations (*SD*_*Pi*_) of the translational and rotational variability were calculated. The group systematic mean ( $\bar{M_{P}}$ , the average of all patients’ means), the systematic error (Σ, the standard deviation around $\bar{M_{P}}$ ) and the random error (*σ*, the root mean square of *SD*_*Pi*_) for translational motions were calculated as:


(1) $$\bar{M_{P}}=\frac{\sum_{i=1}^{N}M_{Pi}}{N}$$

(2) $$\text{Σ}=\sqrt{\frac{\sum_{i=1}^{N}{\left(M_{Pi}-\bar{M_{P}}\right)}^{2}}{N}}$$

(3) $$\sigma =\sqrt{\frac{\sum_{i=1}^{N}SD_{Pi}^{2}}{N}}$$

And the 95% confidence interval of each variable was calculated as follows:


(4) $$CI=\bar{X}\pm Z_{\alpha /2}\cdot \frac{\sigma }{\sqrt{n}}$$

Where $\bar{X}$ is the mean, *Z*_*α*/2_ is the chosen Z-value (1.96 for 95%), *s* is the standard error and *n* is the sample size.

### 2.4 Quantifying the correlations between motions of different DoF

To quantify the correlations between the 6D motions of different DoF, the Pearson’s product-moment correlation coefficient method (54) of each two variables were calculated. Pearson’s correlation coefficient *r* between variables *A* and *B* were calculated using:


(5) $$r=\frac{cov(A,B)}{\sigma_{A}\sigma_{B}}$$

where *cov*(*A*, *B*) = covariance of *A* and *B*; *σ*_*A*_ = population standard deviation of *A*; *σ*_*B*_ = population standard deviation of *B*. *r* can have a value between -1 and 1, where:

-1 indicates a perfectly negative; linear correlation between two variables
- 0 indicates no linear correlation between two variables;
- 1 indicates a perfectly positive linear correlation between two variables;
- |*r*| values within intervals of (0.00,0.19), (0.20,0.39), (0.40,0.59), (0.60,0.79), (0.80,0.99), indicate “very weak”, “weak”, “moderate”, “strong”, and “very strong” correlations, respectively.

### 2.5 CTV-PTV margins calculations based on different published recipes

PTV margins were defined in three separate directions, i.e., LR, AP and SI axes, from the entire patient data using the published recipes on the basis of the population mean, as well as systematic and random errors, as listed in **Table 1**. Among these methods, the recipe 2Σ+0.7*σ* proposed by Stroom (14) and the recipe 2.5Σ+0.7*σ* proposed by Van Herk (49) were most used. In particular, the factor *σ*_*p*_ accounting for dose penumbra width between the dose level selected for dose prescription was included in margin calculation (43, 49, 53). In this study, *σ*_*p*_=2.8 mm was applied based on the analysis of our clinical data. Most recently, margin to attain an expected coverage in 90% of the patient population was defined in three dimensions of LR, AP and SI by (52):


(6) Margin=m±1.28s

where $m=\bar{M_{P}}$ is the population mean and *s*=Σ is the population standard deviation of position variability.

**Table 1:** Margins calculated based on published recipes for target.

No.	References	Recipe	Margin		
			*X* (mm)	*Y* (mm)	*Z* (mm)
1	(46)	0.7σ	0.59	1.22	3.12
2	(47)	1.65σ	1.39	2.87	7.36
3	(14)	2Σ+0.7σ	1.17	2.59	5.62
4	(48)	1.3 Σ±0.5σ	0.80	1.76	3.85
5	(49)	2.5Σ+0.7σ	1.31	2.94	6.24
6	(49)	2.5Σ+1.64(σ–σ*_P_*)	2.08	1.60	5.85
7	(50)	2.5Σ+0.7σ–3	1.01	2.64	5.94
8	(50)	$\sqrt{2.7^{2}{\text{Σ}}^{2}+1.6^{2}\sigma^{2}}-2.8$	0.70	1.94	4.32
9	(10)	$\text{Σ}+\sqrt{\sigma^{2}+{\text{Σ}}^{2}}$	1.18	2.56	5.88
10	(51)	2.5Σ±0.4σ	1.06	2.42	4.91
11	(53)	$2.5\text{Σ}+1.64\sqrt{\sigma^{2}+\sigma_{p}^{2}}-1.64\sigma_{p}$	0.93	2.53	7.17
12	(43)	$2.5\text{Σ}+0.84\sqrt{\sigma^{2}+\sigma_{p}^{2}}-0.84\sigma_{p}$	0.83	2.14	5.19
13	(52)	*m*+1.28*s* m+1.28s	0.93	2.12	4.98
		Mean (mm)	1.08	2.26	5.42
		*SD* (mm)	0.37	0.49	1.17
		Range (mm)	(0.59, 2.08)	(1.22, 2.94)	(3.12, 7.36)
		95% CI (mm)	(0.88, 1.28)	(1.99, 2.53)	(4.78, 6.05)

1.Σ: the standard deviation (SD) of systematic errors;

2.σ: the standard deviation (SD) of random errors;

3.σ_p_: the width of the dose penumbra, and σ_p_=2.8 mm was used here;

4.m: the population mean of position variability;

5.s: the population standard deviation of position variability, and s=Σ.

## 3 Results

### 3.1 Tumor motions and population errors

**Figure 2:**
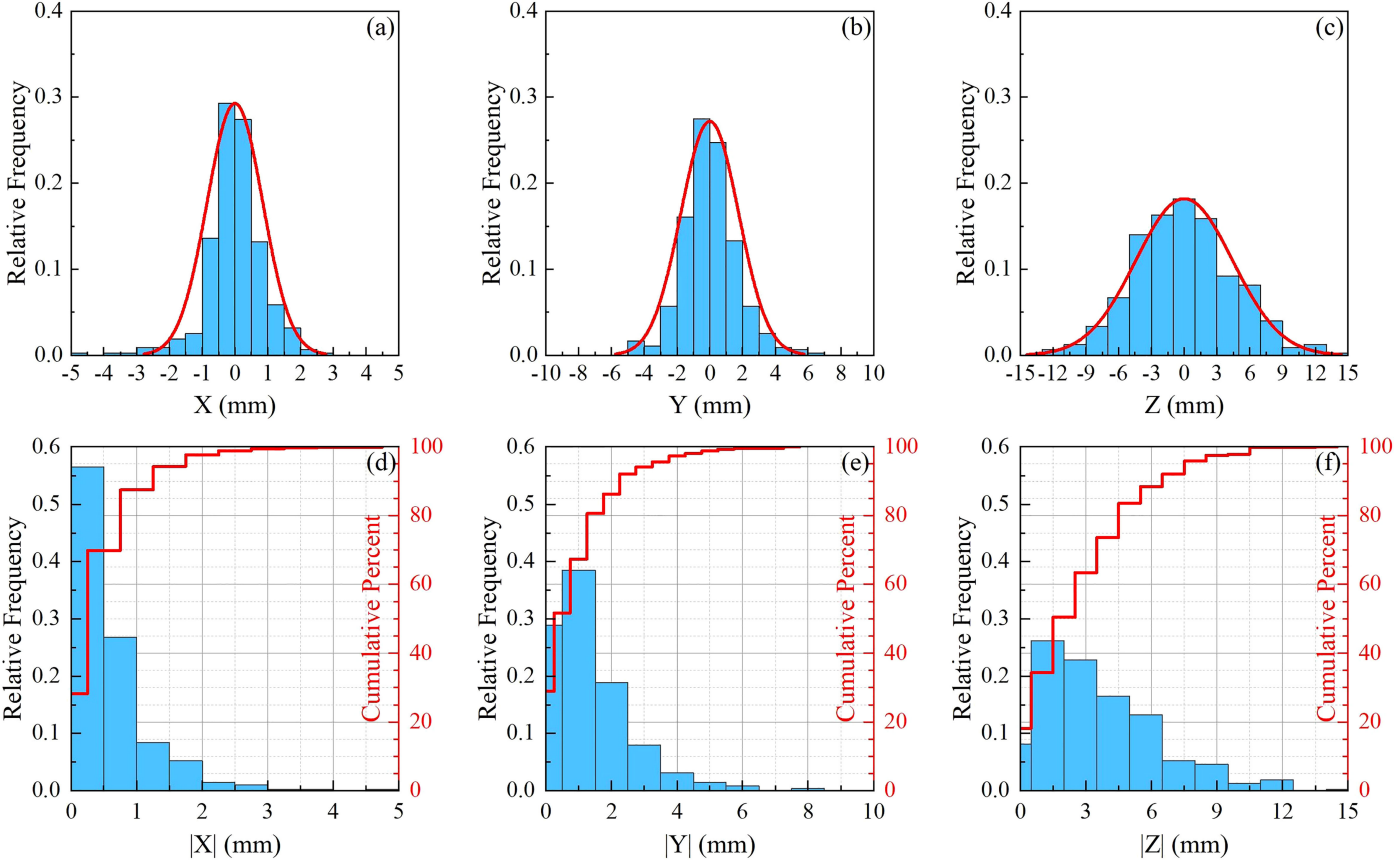
Statistics for translational movements. **(A–C)** are normalized frequency histograms of translational movements in the LR (*X* ), AP (*Y* ) and SI (*Z* ) axes, respectively. The red curves are a Gaussian function fitting for the histograms. **(D–F)** are normalized histograms (blue bars) and cumulative percent distribution (red histograms) with different absolute motion magnitudes for LR, AP and SI directions.

**Figure 3:**
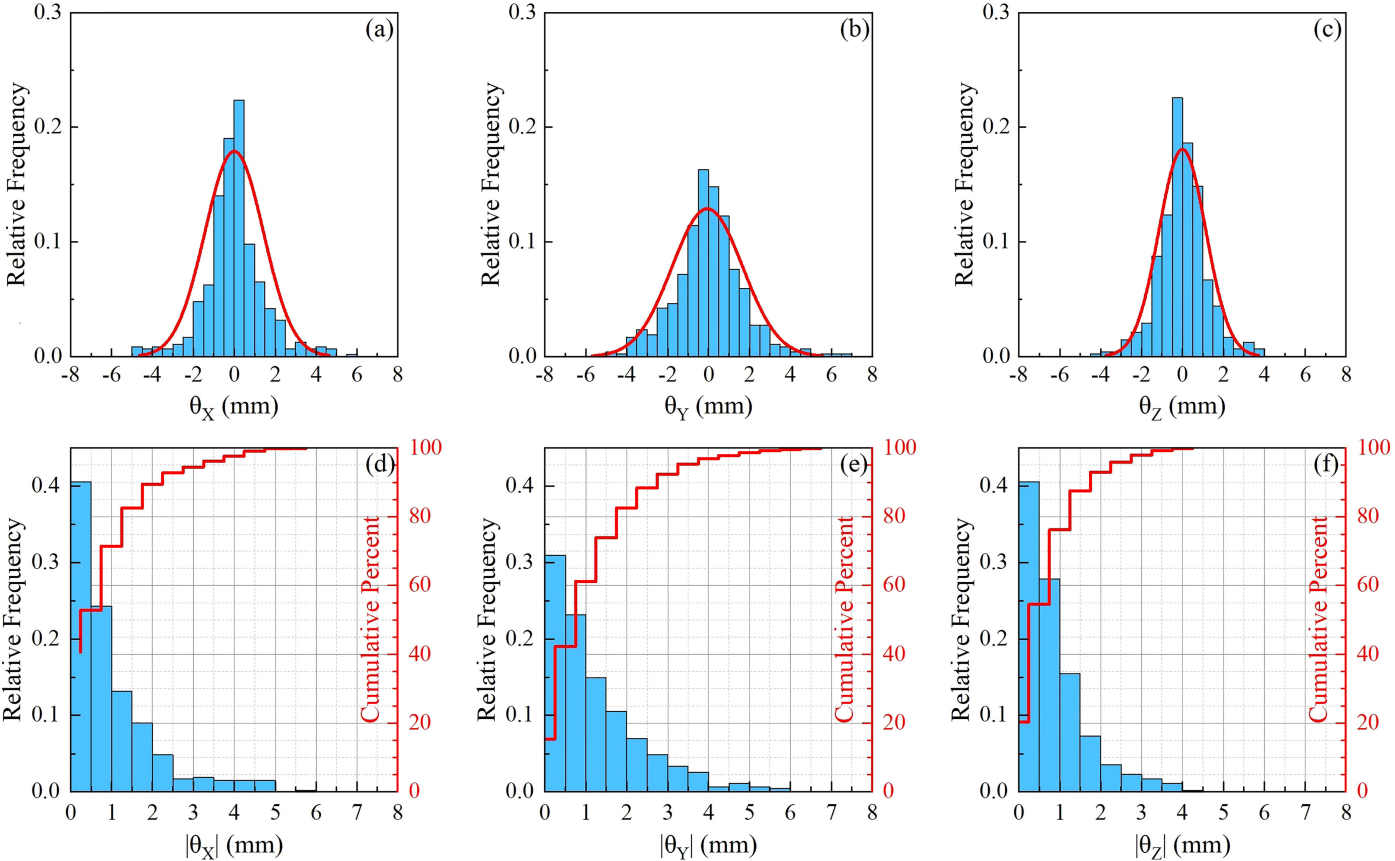
Statistics for rotational movements. **(A–C)** are normalized frequency histograms of rotational movements around LR (*θ*_*X*_ ), AP (*θ*_*Y*_ ) and SI (*θ*_*Z*_ ) axes, respectively. The red curves are a Gaussian function fitting for the histograms. **(D–F)** are normalized histograms (blue bars) and cumulative percent distribution (red histograms) with different absolute rotation angles around LR, AP and SI axes.

A total of 495 pairs of orthogonal images were retrospectively analyzed for 12 patients. The translational and rotational motions were analyzed for each patient and the whole sample. **Figures 2A–C** are normalized frequency histograms of translational movements in the LR (*X*), AP (*Y*) and SI (*Z*) axes, respectively. The red curves are Gaussian function fitting for the histograms. **Figures 2D–F** are normalized histograms (blue bars) and cumulative percent distribution (red histograms) with different absolute motion magnitudes for LR, AP and SI directions. It indicated that the translational motions for 95% of the samples were not larger than 2.0 mm, 3.5 mm and8.5 mm in LR, AP and SI directions, respectively. The similar histograms for rotational angles around LR(*θ*_*X*_ ), AP(*θ*_*Y*_ ) and SI(*θ*_*Z*_ ) axes were displayed in **Figure 3**, which indicated that the rotation angles for 95% of the samples were not larger than 3.0^∘^ , 3.5^∘^ and 2.5^∘^ for *θ*_*X*_ , *θ*_*Y*_ and *θ*_*Z*_ , respectively.

The statistics of motion magnitudes were summarized in **Table 2** in terms of mean ± SD. The average (range) translational motion amplitudes were 0.56 (0.22–1.14), 1.24 (0.62–2.65), and 3.38 (1.36–5.46) mm in the LR, AP, and SI directions, respectively. Translational motion range in 3D space (*R*) was 3.98 (1.61-6.18) mm. The population systematic (Σ ) errors were 0.29, 0.69 and 1.25 mm, and the population random (*σ* ) errors were 0.84, 1.74 and 4.46 for *X, Y*, and *Z*, respectively. It should be stressed that the effect of random error is 2.5 to 3.5 times more important than the effect of system error. These population errors in different directions would be used for PTV margin calculations. The rotational angles in LR (*þeta*_*X*_ ), AP (*θ*_*Y*_ ), and SI (*θ*_*Z*_ ) directions were 0.88±0.58^∘^ , 1.24±0.53^∘^ , and 1.12±1.06^∘^ , respectively. From **Table 2** and **Figures 2**, **3**, we could identify that translation in X direction was the smallest and Z was the biggest, rotation in *θX* was the smallest and *θY* was the biggest.

**Table 2:** Systematic and random errors of translation and rotation in different directions.

Pat.	Fid.	Imag.	*X* (mm)	*Y* (mm)	*Z* (mm)	*R* (mm)	*θ_X_* (°C)	*θ_Y_* (°C)	*θ_Z_* (°C)
1	3	43	0.79±1.02	0.72±0.91	2.28±2.78	2.75±1.44	1.83±2.37	2.05±2.52	1.08±1.34
2	3	40	0.22±0.29	0.61±0.85	1.36±1.85	1.61±1.28	0.52±0.67	0.43±0.55	0.25±0.32
3	3	25	0.82±1.26	0.62±0.75	3.78±4.42	3.98±2.42	0.27±0.31	0.79±1.09	0.47±0.53
4	3	61	0.80±1.02	1.76±2.15	2.70±3.34	3.65±2.17	1.55±1.97	1.86±2.13	0.85±1.13
5	3	40	0.33±0.77	0.62±1.20	2.00±4.93	4.47±2.53	0.60±0.84	1.12±1.28	1.23±1.66
6	3	58	0.48±0.63	0.96±1.21	4.53±5.25	4.80±2.67	0.60±0.77	1.94±2.52	0.91±1.18
7	3	46	1.14±1.44	2.65±3.35	4.79±5.98	5.69±4.09	1.80±2.21	1.73±2.21	1.09±1.31
8	3	43	0.41±0.50	1.14±1.40	3.55±4.31	3.82±2.49	1.48±1.76	0.66±0.73	0.35±0.41
9	4	31	0.45±0.54	1.09±1.31	3.65±4.37	3.93±2.38	0.73±0.84	1.16±1.43	0.86±1.12
10	4	26	0.79±1.01	2.50±2.86	5.46±6.26	6.18±3.19	0.47±0.60	1.04±1.24	1.08±1.43
11	4	32	0.21±0.24	0.77±0.98	2.01±2.55	2.22±1.62	0.48±0.56	0.70±0.86	0.74±0.90
12	4	33	0.23±0.31	1.44±1.65	4.47±5.12	4.72±2.60	0.23±0.66	1.44±0.54	4.47±1.46
Population $\bar{M_{P}}$			0.56	1.24	3.38	3.98	0.88	1.24	1.12
PopulationΣ			0.29	0.69	1.25	1.28	0.58	0.53	1.06
Populationσ			0.84	1.74	4.46	2.52	1.33	1.59	1.14

### 3.2 Correlations between motions of different DoFs

**Figure 4:**
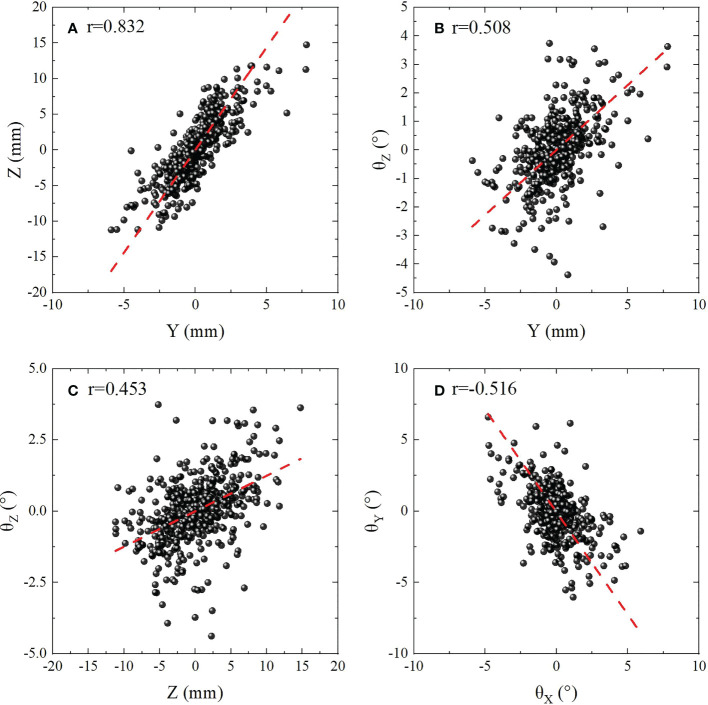
Scatter plot showing the correlation between six dimensional movements with |*r*|>0.4 . **(A)** (*Z*,*Y* ) with *r*=0.832 for indicates strong correlation. **(B–D)** (*Y*,*θ*_*Z*_ ), (Z, *θ*_*Z*_ ) and (*θ*_*X*_,*θ*_*Y*_ ) with 0.4<|*r*|<0.6 indicates medium correlations. The red dashed lines are linear fitting.

Correlation coefficients between the 6D motions with different DoFs were analyzed with Pearson’s product-moment correlation coefficient method, and a total of 15 Pearson’s correlation coefficients were calculated. As shown in **Table 3**, it could be identified that weak correlations were observed for (*X*, *Y*), (*X*, *Z*), and (*θ*_*Y*_,*θ*_*Z*_ ). (*Y*,*θ*_*Z*_ ), (*Z*,*θ*_*Z*_ ) and (*θ*_*X*_,*θ*_*Y*_ ) had medium correlations (0.4<|*r*|<0.6 ). The scatter plot in **Figure 4** showed the correlation between six dimensional movements with |*r*|>0.4 ,which were also displayed with bold font in **Table 3**. **Figures 4A–D** were (*Z*, *Y*), (*Y*,*θ*_*Z*_ ), (Z, *θ*_*Z*_ ) and (*θ*_*X*_,*θ*_*Y*_ ) with *r*=0.832 , 0.508, 0.453 and -0.516, respectively. Specially, strong correlation was obtained for (*Y, Z*) with *r*=0.832 . The red dashed lines in the figure are linear fitting.

**Table 3:** Pearson’s correlation coefficient between six dimensional movements of translation and rotation.

		*X*	*Y*	*Z*	*θ_X_*	*θ_Y_*
*Y*	*r*	0.273				
	*p*	0.000				
*Z*	*r*	0.261	**0.832**			
	*p*	0.000	**0.000**			
*θ_X_*	*r*	0.122	-0.141	-0.161		
	*p*	0.007	0.002	0.000		
*θ_Y_*	*r*	0.024	-0.087	-0.094	**-0.516**	
	*p*	0.596	0.059	0.039	**0.000**	
*θ_Z_*	*r*	-0.039	**0.508**	**0.453**	-0.108	-0.366
	*p*	0.396	**0.000**	**0.000**	0.018	0.000

### 3.3 Margins calculated based on published recipes

**Figure 5:**
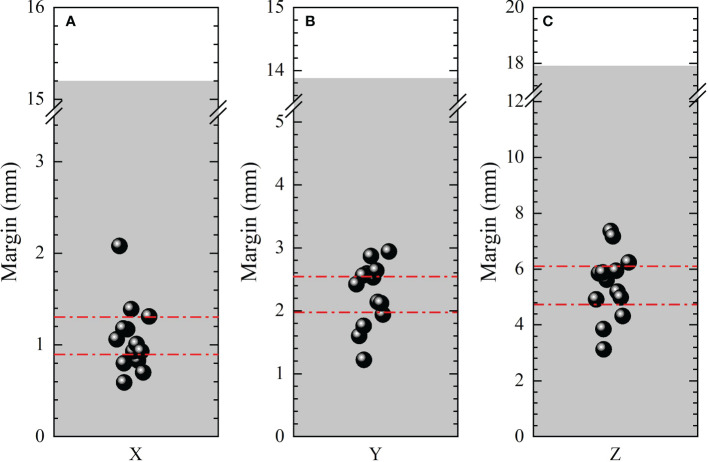
Comparison of anisotropic PTV margins in LR (*X* ), AP(*Y* ) and SI (*Z* ) directions between this work and that from M. Sharma’s work. The black solid balls and red dashed lines represent the PTV margins and related 95% confident intervals obtained in this work. The gray areas indicate the range of margins from M. Sharma’s work (55).

Different published recipes and margins calculated based on published recipes for target were shown in **Table 1**. The average margins based on all the recipes listed in **Table 1** were 1.08±0.37 , 2.26±0.49 , 5.42±1.17 , with ranges of (0.59,2.08), (1.22,2.94) and (3.12,7.36) in LR, AP and SI directions, respectively. And the 95% confidence intervals for anisotropic margins were (0.88,1.28), (1.99,2.53), and (4.78,6.05), respectively. Among all recipes, the maximum margins of 2.08, 2.94 and 7.36 mm were obtained in LR, AP and SI directions based on the recipes 2.5Σ+1.64(*σ*−*σ*_*p*_) , 2.5Σ+0.7*σ* , and 1.65*σ* , respectively. Recently, a systematic review and meta-analysis for liver SBRT with different motion management strategies was published by Sharma et al. (55). The margins obtained in this study were compared with those from Sharma’s work with free-breathing, as was displayed in **Figure 5** and subfigure A, B and C represents PTV margins in X, Y and Z directions,respectively. The black solid balls and red dashed lines represent the PTV margins and related 95% confidence intervals obtained in this work. The gray areas indicate the range of margins from M. Sharma’s work (55). The margins obtained in this study are much smaller than the results of Sharma, but are within the range of standard deviation.

## 4 Discussion

This study was mainly focused on three points: quantifying the tumor motion variability, analyzing the correlations between different DoFs, and estimating PTV margins based on published recipes to provide potential recommendations for PTV margins for liver SBRT with fiducial tracking technique. In particular, the correlation coefficients between motions of different DoF were rarely investigated in the past. Since most IGRT techniques are not able to quantify rotational motions, only translational motions were taken into account for PTV margins. While conducting stereotactic radiotherapy on CK system, the advantage of TLS could execute rotational correction in real-time and the mean residual errors of translational motions will be reduced by 45% with rotational correction which was demonstrated in previous study (34). Less translational correction was needed in other words smaller margin was needed. We summarized different published recipes and calculated target margins based on them. Our results may provide recommendations for target margins in those patients without fiducials implanting. The correlations discovered in this study would be potentially used to estimate tumor rotations without fiducial implanting.

Our study indicated that the liver motion range changes of 1.08 mm LR, 2.26 mm AP, and 5.42 mm SI during SBRT were smaller in the majority of this patient population, when compared with Sharma’s work (55) of 4.2, 5.4 and 9.7 mm, respectively. However, comparable margins of 1-3 mm AP/LR and 3-6 mm SI were also reported by Yang and Shimohigashi (44, 56). It is essential to notice that these margins are organization-specific, even though the methodology is commonly applied. The derived PTV margins can be used as an orientation for other radiotherapy organizations with a range of protocols of practice for their IGRT system. However, PTV margins should always be decided by different organizations, on the groundwork of the particular experience of different organizations. Ideally, margins have to be personalized for every patient comprising the setup variability, motion uncertainty, and precision during treatment. Most of the researchers reported that in AP and SI directions,tumor motion is larger than that in LR direction, which is consistent with this study. Furthermore, it is vital to consider that population-based margins do not accurately characterize the necessary margins for personal patients. Based on population-based studies, even though different advice for crucial margins were supplied by many authors, the real extent of CTV–PTV margins is also institute-specific. These margins should be checked specially when applying a new technique and/or assessing new immobilization devices.

Systematic and random errors have different effects on dose distribution. Systematic errors are persistent and consistent during the treatment period, nevertheless random errors anticipate a different magnitude and direction for each treatment fraction. Where systematic errors lead to the shift of the dose distribution, random errors result in blurring. With regard to systematic errors, all fractions are equally affected which could result in very serious trouble due to the shifting of the dose distribution, as the CTV may shift out of the high-dose region. However, random error may come up every day and small dose change will lead to blurring causing reduce of the dose at the high-dose region close to the edge.

A vital disadvantage of these margin recipes is they are lacking of adequately incorporating both rotational and translational errors. Another point that used to be out of the scope of this study but worth mentioning is the impact of liver tumor location and other elements that could have an impact on the position variability. The liver tumors position variability has a significant dependence on the vicinity of tumor in the segments. For example, tumors situated in peripheral segments have a tendency to experience increased intra-fraction movement relative to these within centrally located segments (32). Finally, the presented consequences do not include the target delineation uncertainties. This is out of the scope of this research because we have centered on margins due to inter- and intra-fraction motion at the treatment period. The extra margin to account for target delineation to be included in the overall margin needs to be studied locally.

## 5 Conclusion

In this study, the rotational and translational motions of tumors during liver SBRT based on fiducial tracking of the CyberKnife system were estimated *via* the ICP algorithm. Tumor motion was greatest in the SI direction. Interestingly, medium and strong correlations were obtained between *Y*-*Z*, *Y* - *θ*_*Z*_ , *Z* - *θ*_*Z*_ and *θ*_*X*_ -*θ*_*Y*_ . The PTV margins calculated in this study were consistent with some previous works, and treatment site-specific anisotropic margins of 2, 3 and 7 mm in LR, AP, and SI directions were recommended to compensate for intra-fractional tumor motions to systematic and random errors.

## Data availability statement

The original contributions presented in the study are included in the article/**Supplementary Material**. Further inquiries can be directed to the corresponding author.

## Ethics statement

We state that the studies involving human participants were reviewed and approved by the institutional review board of National Cancer Center/National Clinical Research Center for Cancer/Cancer Hospital & Shenzhen Hospital. Written informed consent to participate in this study was provided by the participants. We confirm that all methods were carried out in accordance with relevant guidelines and regulations.

## Author contributions

XS: participation in the whole work; generating treatment plans; drafting of the manuscript; data analysis; final approval of the submitted version; ZD: participation in the whole work; perception and design; drafting of the manuscript; data analysis; final approval of the submitted version; MX: data analysis and drafting of the manuscript; XG: drafting and final approval of the submitted version; HS: data analysis; YL: data analysis. All authors contributed to the article and approved the submitted version.

## Funding

This study was supported by Youth Start-up Fund of Shenzhen Cancer Hospital (E010321021), Shenzhen Postdoctoral Research Funds (25005), Basic and Applied Basic Research Foundation of Guangdong Province (2020A1515110335), Sanming Project of Medicine in Shenzhen (SZSM201612063) and Shenzhen Key Medical Discipline Construction Fund (SZXK013).

## Conflict of interest

The authors declare that the research was conducted in the absence of any commercial or financial relationships that could be construed as a potential conflict of interest.

## Publisher’s note

All claims expressed in this article are solely those of the authors and do not necessarily represent those of their affiliated organizations, or those of the publisher, the editors and the reviewers. Any product that may be evaluated in this article, or claim that may be made by its manufacturer, is not guaranteed or endorsed by the publisher.
