# Supplementary material for: Quantifying 6D tumor motion and calculating PTV margins during liver stereotactic radiotherapy with fiducial tracking
Source: Front Oncol. 2022 Nov 16;12:1021119. doi: 10.3389/fonc.2022.1021119 (PMC9709257; doi:10.3389/fonc.2022.1021119)
Supplement: Supplementary file 1 [file DataSheet_1.pdf]

Supplementary Material

1 GENERAL INTRODUCTION OF THE ITERATIVE CLOSEST POINT (ICP) ALGORITHM

The iterative closest point (ICP) algorithm, originally proposed by (Besl and McKay, 1992), is one of the most popular methods used for estimating the rigid transformation of roughly aligned 3D data sets. It is supposed that two point sets $\mathbf{X}$, $\mathbf{Y} \in R^K$,
the reference point set $\mathbf{X} = \{\mathbf{x_i}, i = 1, 2, ..., N\}$ with $\mathbf{x_i} = [x_{il}, ..., x_{iK}]^T$,
the target point set $\mathbf{Y} = \left\{\mathbf{y_j}, i = 1, 2, ..., M\right\}$ with $\mathbf{y_j} = \left[y_{jl}, ..., y_{jK}\right]^T$,
the closest point set $\mathbf{Z} = \left\{\mathbf{z_j}, i = 1, 2, ..., M\right\}$ with $\mathbf{z_j} = \left[z_{jl}, ..., z_{jK}\right]^T$, and $\mathbf{z_j} \in \mathbf{X}$.
The aim of the ICP algorithm is to find a rigid transformation $(\mathbf{R_0}, \mathbf{T_0})$ to minimize the mean square sum of the Euclidean distances between the target point set $\mathbf{Y}$ transformed by $(\mathbf{R_0}, \mathbf{T_0})$ and its closest point set $\mathbf{Z}$ in $\mathbf{X}$, in which $(\mathbf{R_0}$ is a $K \times K$ rotation matrix and $\mathbf{T_0})$ is a $K \times 1$ translation matrix.

$$\mathbf{T} = \begin{bmatrix} t_1 \\ t_2 \\ t_3 \end{bmatrix} \tag{S1}$$

$$\mathbf{R_0} = \begin{bmatrix} r_{11} & r_{12} & r_{13} \\ r_{21} & r_{22} & r_{23} \\ r_{31} & r_{32} & r_{33} \end{bmatrix} \tag{S2}$$

which satisfies $\mathbf{R_0}\mathbf{R_0}^T = \mathbf{I}$, with $\mathbf{I}$ being a unit matrix. And accordingly, the objection function to be minimized is

$$J\left(\mathbf{R_0}, \mathbf{T_0}\right) = Min\left\{\frac{1}{M}\sum_{i=1}^{M}\|\mathbf{z_i} - (\mathbf{R_0}\cdot\mathbf{y_i} + \mathbf{T_0})\|\right\} \tag{S3}$$

The centroids of the target point set $\mathbf{Y}$ and the closest point set $\mathbf{Z}$ are given by

$$\begin{cases} \overline{\mathbf{y}} = \frac{1}{M}\sum\limits_{i=1}^{M}\mathbf{y_i} \\ \overline{\mathbf{z}} = \frac{1}{M}\sum\limits_{i=1}^{M}\mathbf{z_i} \end{cases} \tag{S4}$$

The cross-covariance matrix $\mathbf{Cov}\left(\mathbf{Y}, \mathbf{Z}\right)$ of the point sets $\mathbf{Y}$ and $\mathbf{Z}$ is given by

$$\begin{aligned} \mathbf{Cov}(\mathbf{Y}, \mathbf{Z}) =& \frac{1}{M}\sum_{i=1}^{M}\left[\left(\mathbf{y_i} - \overline{\mathbf{y}}\right)\left(\mathbf{z_i} - \overline{\mathbf{z}}\right)\right]^T \\ =& \frac{1}{M}\sum_{i=1}^{M}\mathbf{y_i}\mathbf{z_i}^T - \overline{\mathbf{y}\mathbf{z}}^T \end{aligned} \tag{S5}$$

The cyclic components of the anti-symmetric matrix B_{ij} of $\mathbf{Cov}\,(\mathbf{Y}, \mathbf{Z})$ is expressed by

$$B_{ij} = \left(\mathbf{Cov}\,(\mathbf{Y}, \mathbf{Z}) - \mathbf{Cov}\,(\mathbf{Y}, \mathbf{Z})^T\right)_{ij} \tag{S6}$$

B_{ij} is used to construct the column vector $\mathbf{\Omega}$ shown by

$$\mathbf{\Omega} = \begin{bmatrix} B_{23} & B_{31} & B_{12} \end{bmatrix}^T \tag{S7}$$

Further, $\mathbf{X}$ is applied to yield a symmetric 4×4 $\mathbf{\Sigma}\,(\mathbf{Cov}\,(\mathbf{Y}, \mathbf{Z}))$,

$$\mathbf{\Omega}\,(\mathbf{Cov}\,(\mathbf{Y}, \mathbf{Z})) = \begin{bmatrix} tr\,(\mathbf{Cov}\,(\mathbf{Y}, \mathbf{Z})) & \mathbf{\Omega}^T \\ \mathbf{\Omega} & \mathbf{Cov}\,(\mathbf{Y}, \mathbf{Z}) + \mathbf{Cov}\,(\mathbf{Y}, \mathbf{Z})^T - tr\,(\mathbf{Cov}\,(\mathbf{Y}, \mathbf{Z}))\,\mathbf{I}_{3\times3} \end{bmatrix} \tag{S8}$$

where $tr\,(\mathbf{A})$ denotes the trace of matrix $\mathbf{A}$, and $\mathbf{I}_{3\times3}$ is a 3×3 identity matrix. The unit eigenvector $\hat{\mathbf{q}} = q_0\mathbf{i} + q_1\mathbf{j} + q_2\mathbf{k} + q_3 = \begin{bmatrix} q_0 & q_1 & q_2 & q_3 \end{bmatrix}^T$ for $q_0^2 + q_1^2 + q_2^2 + q_3^2 = 1$ corresponding to the maximum eigenvalue of the matrix $\mathbf{\Sigma}\,(\mathbf{Cov}\,(\mathbf{Y}, \mathbf{Z}))$ is referred to as the rotation represented by the unit quaternion. According to $\mathbf{q}$, the rotation matrix $\mathbf{R_0}$ is obtained by

$$\mathbf{R_0} = \begin{bmatrix} q_0^2 + q_1^2 - q_2^2 + q_3^2 & 2\,(q_1q_2 - q_0q_3) & 2\,(q_1q_3 - q_0q_2) \\ 2\,(q_1q_2 + q_0q_3) & q_0^2 - q_1^2 + q_2^2 + q_3^2 & 2\,(q_2q_3 - q_0q_1) \\ 2\,(q_1q_3 - q_0q_2) & 2\,(q_2q_3 - q_0q_1) & q_0^2 - q_1^2 - q_2^2 + q_3^2 \end{bmatrix} \tag{S9}$$

The quaternion representation could be transformed to the representation respected to Euler angle θ and the Euler axis $\hat{\mathbf{e}} = \begin{bmatrix} e_x & e_y & e_z \end{bmatrix}^T$ with the following relationships:

$$\begin{cases} q_0 = e_x \sin\theta/2 \\ q_1 = e_y \sin\theta/2 \\ q_2 = e_z \sin\theta/2 \\ q_3 = \cos\theta/2 \end{cases} \tag{S10}$$

By bring EquationS10 into EquationS9, we can get

$$\mathbf{R_0} = \begin{bmatrix} sin^2\frac{\theta}{2}(e_x^2 + e_y^2 - e_z^2) + \cos^2\frac{\theta}{2} & 2\sin\frac{\theta}{2}(e_ye_z - e_x\cos\frac{\theta}{2}) & 2\sin\frac{\theta}{2}(e_y\cos\frac{\theta}{2} - e_xe_z\sin\frac{\theta}{2}) \\ 2sin^2\frac{\theta}{2}(e_ye_zsin\frac{\theta}{2} + e_xcos\frac{\theta}{2}) & sin^2\frac{\theta}{2}(e_x^2 - e_y^2 + e_z^2) + \cos^2\frac{\theta}{2} & 2sin\frac{\theta}{2}(e_z\cos\frac{\theta}{2} - e_xe_ysin\frac{\theta}{2}) \\ 2\sin\frac{\theta}{2}(e_y\cos\frac{\theta}{2} - e_xe_z\sin\frac{\theta}{2}) & 2sin\frac{\theta}{2}(e_z\cos\frac{\theta}{2} - e_xe_ysin\frac{\theta}{2}) & \sin^2\frac{\theta}{2}(e_x^2 - e_y^2 - e_z^2) + \cos^2\frac{\theta}{2} \end{bmatrix} \tag{S11}$$

Then the translation matrix $\mathbf{T_0}$ is

$$\mathbf{T_0} = \overline{\mathbf{z}} - \mathbf{R_0} \cdot \overline{\mathbf{y}} \tag{S12}$$

REFERENCES

Besl, P. and McKay, H. (1992). A method for registration of 3-d shapes. *IEEE Transactions on Pattern Analysis and Machine Intelligence* 14, 239–256. doi:https://doi.org/10.1109/34.121791
